# Supplementary material for: Public engagement for the conduct of a controlled human infection study testing vaccines against Necator americanus (hookworm) in areas of active hookworm transmission in Brazil
Source: PLoS One. 2024 Jun 3;19(6):e0299022. doi: 10.1371/journal.pone.0299022 (PMC11146735; doi:10.1371/journal.pone.0299022)
Supplement: Attachment — Submitted filename: Public Engagement Manuscript.pdf [file pone.0299022.s002.pdf]

Francis Balungnaa D. Veriegh (PhD)
Dept of Biological Science
School of Sciences
University of Energy and Natural Resources (UENR)
P. O. Box 214, Sunyani, Ghana

October 17, 2023

The Editor
Committee for Human Research and Ethics
University of Energy and Natural Resources

Dear Sir/Madam,

RE: INVITATION TO ACT AS A PEER REVIEWER

This report is submitted in response to your request to peer review the underlisted manuscript's suitability for publication in PLoS ONE journal.

Manuscript Number: PONE-D-23-25173
Manuscript Title: Public Engagement for the Conduct of a Controlled Human Infection Study testing vaccines against Necator americanus (hookworm) in areas of active hookworm transmission in Brazil.
Authors: Luciene Barra Ribeiro. Andréa Gazzinelli. Helton da Costa Santiago. Jacqueline Araújo Fiuza. Lucas Lobato. Rodrigo Correa de Oliveira. Maria Flávia Gazzinelli Bethony.
Article type: Research Article

The manuscript satisfies all the seven (7) criteria for publication in the PLOS ONE journal, however, the following comments and suggestions below are for the authors' consideration.

The statistical analysis performed was not appropriate and rigorous because even though the researchers used Bardin's Content Analysis for their data analysis but failed to categorize or codify the words, themes, and concepts within the text that would enable them to subject their data to quantitative analysis. Content analysis can be both quantitative (focused on counting and measuring) and qualitative (focused on interpreting and understanding). In both types, you categorize or "code" words, themes, and concepts within the texts and then analyze the results. It is therefore recommended that the authors do the needful and subject their data to quantitative analysis.

Line 4: make 1 attached to author Andréa Gazzinelli superscript

Line 24: Delete the definite article "the" that precedes ongoing natural transmission

Line 26: Add "is" to before the first word "targeted"

Line 49: Change the word "has" that precedes already to "have"

Line 53: Delete phrase "have been conducted" for repetition in line 49 and 50

Line 59: Change participation to participants and delete "in" that precedes areas

Line 64: Replace CHOMS with CHIS

Line 65: Replace the word "now" with "which"

Francis Balungnaa D. Veriegh (PhD)
Dept of Biological Science
School of Sciences
University of Energy and Natural Resources (UENR)
P. O. Box 214, Sunyani, Ghana

Line 78: Replace “resulting” with “results”

Line 108: Replace the word “among” with “from”

Line 125: Define TCLE before you abbreviate it

Line 126: Define the age limits of minors in Americaninhas and re-caste the last sentence (e.g. This study did not include minors).

Line 127: Which body promulgated resolution 466 of 2012?

Line 175: Separate goodwill into “good” and “will”

Line 216: Highlighted

Line 346: Re-caste (i.e. residents' perceptions of CHIS in an endemic area)

Line 354: Delete “thought”

Line 358: Replace “with” with “which”

Re-draw figs. 1 and 2 to show at least the North Pole, and scale.

Thank you.

Yours faithfully,

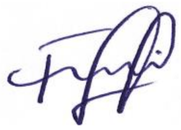

Dr. Francis B. D. Veriegh
